# Cine DENSE MRI of mechanical activation in heart failure patients referred for cardiac resynchronization therapy

**DOI:** 10.1186/1532-429X-18-S1-P215

**Published:** 2016-01-27

**Authors:** Daniel A Auger, Sophia X Cui, Xiao Chen, Jorge A Gonzalez, Christopher M Kramer, Kenneth C Bilchick, Frederick H Epstein

**Affiliations:** 1 Biomedical Engineering, University of Virginia, Charlottesville, VA USA; 2 Medical Solutions, Siemens, Princeton, NJ USA; 3 Medicine, Cardiovascular Medicine, University of Virginia, Charlottesville, VA USA; 4 Radiology and Medical Imaging, University of Virginia, Charlottesville, VA USA

## Background

In patients with heart failure (HF) and left bundle branch block (LBBB) cardiac resynchronization therapy (CRT) improves left ventricular (LV) function and leads to reverse LV remodeling. A major concern with CRT remains that using current clinical indicators and implementation methods, 30-40% of patients are non-responders. CRT response is highly dependent on the presence of delayed mechanical activation and absence of scar at the LV lead implantation site [1]. The aims of this study were to establish the correlation between mechanical activation time measured by cine DENSE MRI and electrical activation time (QLV) at the LV lead implantation site, and to characterize the heterogeneity of the location of the site of the latest mechanically activated segment in patients with HF-LBBB. Furthermore, we evaluated the frequency in which CRT leads are placed in suboptimal regions.

## Methods

**Figure 1:**
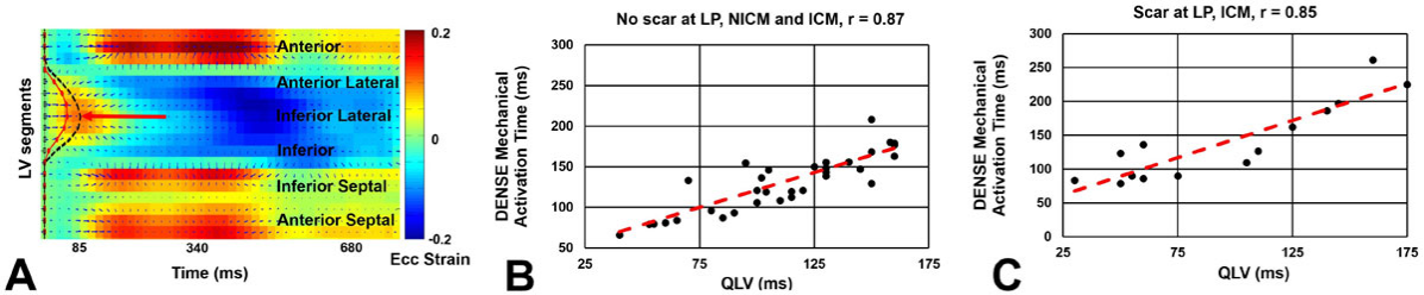
**(A) An active contour applied to a strain matrix automatically depicts the region of late mechanical activation (red arrow)**. (B) Correlation plot between DENSE mechanical activation time and electrical activation time (QLV) for segments where the CRT LV lead was placed in a region without scar. (C) Correlation plot between DENSE mechanical activation time and QLV for segments where the CRT LV lead was placed in a region with scar

Short axis images were acquired on a 1.5T MR system from 6 healthy subjects and 50 HF-LBBB patients (27 with myocardial scar and 23 without scar) with established clinical criteria for CRT. Late gadolinium enhancement (LGE) was used to detect the scar location. DENSE circumferential strain was computed using previously described methods [2, 3]. Spatiotemporal strain data were arranged into a 2D matrix over space (LV segment) and time (cardiac phase) and singular value decomposition (SVD) was used to denoise the strain data. An active contour method automatically detected regions of delayed mechanical activation, defined by the time-of-onset of contraction, as per Figure 1(A). Mechanical activation time was correlated with QLV which was recorded during the CRT procedure.

## Results

**Figure 2:**
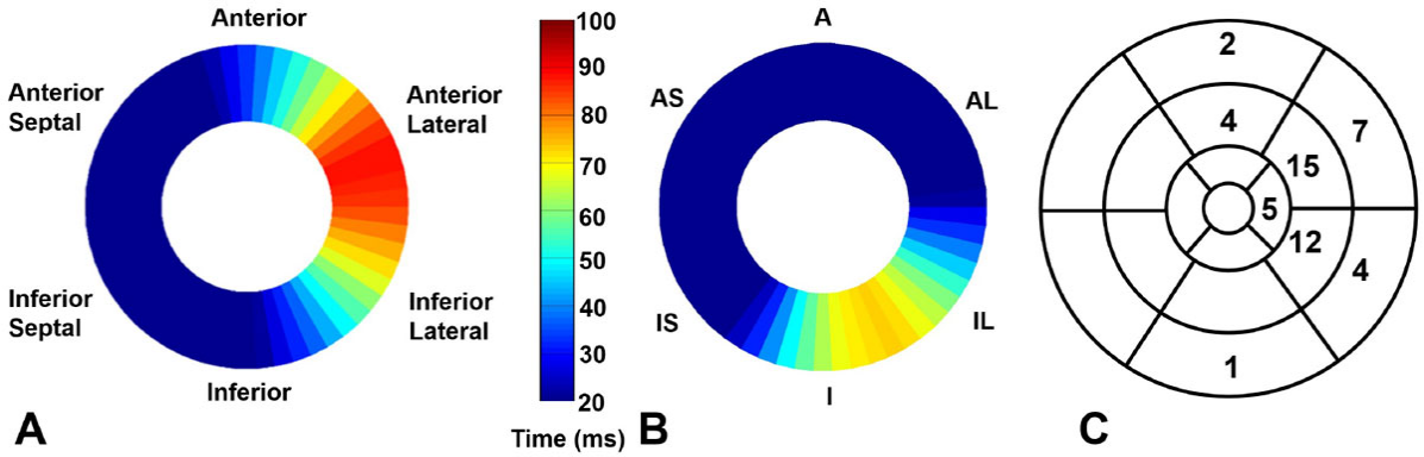
**Mid-ventricular DENSE mechanical activation time maps for patients with LBBB and no scar depicting a mechanical delay in the anterior lateral wall of the LV (A) and a mechanical delay in the inferior lateral wall of the LV (B)**. (C) Bulls-eye plot showing the heterogeneity of the location of the site of latest mechanical activation for all 50 patients

Figure 1(B, C) shows good correlations between DENSE mechanical activation time and QLV at matched locations in the LV for patients without and with scar, with a greater electrical-mechanical delay in regions with scar Figure 1(C). Figure 2(A, B) illustrates the variation in the location of the latest mechanically activated segment in 2 patients with HF-LBBB, and the overall heterogeneity of the site of the latest mechanically activated segment is shown in a bulls-eye plot in Figure 2(C) for all patients. Using standard CRT methods, in 55% of patients the LV lead was placed in an area with less than 80% of the maximal delay, and in 30% of patients the LV lead was placed in a region with less than 60% of the maximal mechanical delay.

## Conclusions

In HF patients selected for CRT, mechanical activation times measured with cine DENSE correlate well with electrical activation times. Imaging of mechanical activation demonstrates heterogeneity of the site of latest activation. Based on the heterogeneity and the suboptimal lead locations achieved using current CRT methods, pre-procedure MRI of mechanical activation shows potential for improving the implementation of CRT.
